# The ecosystem approach to health is a promising strategy in international development: lessons from Japan and Laos

**DOI:** 10.1186/s12992-015-0093-0

**Published:** 2015-02-16

**Authors:** Takashi Asakura, Hein Mallee, Sachi Tomokawa, Kazuhiko Moji, Jun Kobayashi

**Affiliations:** Department of Education, Tokyo Gakugei University, 4-1-1 Nukuikita, Koganei, 184-8501 Tokyo Japan; Research Institute for Humanity and Nature, 457-4 Motoyama, Kamigamo, Kita, Kyoto, 603-8047 Japan; Department of Education, Shinshu University, 6-Ro, Nishinagano, Nagano, 380-8544 Japan; Graduate School of International Health Development, Nagasaki University, 1-12-4 Sakamoto, Nagasaki, 852-8523 Japan; Faculty of Medicine, University of The Ryukyus, 207 Aza Uehara, Nishihara-cho, Okinawa, 903-0215 Japan

**Keywords:** Ecosystem approach, Ecohealth, Ecohealth education, Developing countries, Ecosystem degradation, Health consequences, Development and health, Laos, Japan

## Abstract

**Background:**

An ecological perspective was prominently present in the health promotion movement in the 1980s, but this seems to have faded. The burden of disease the developing world is facing cannot be addressed solely by reductionist approaches. Holistic approaches are called for that recognize the fundamentally interdependent nature of health and other societal, developmental, and ecosystem related factors in human communities. An ecosystem approach to human health (ecohealth) provides a good starting point to explore these interdependencies.

**Discussion:**

Development assistance is often based on the assumption that developed countries can serve as models for developing ones. Japan has provided lavish assistance to Laos for example, much of it going to the development of transport networks. However, there is little sign that there is an awareness of the potentially negative environmental and health impacts of this assistance. We argue that the health consequences of environmental degradation are not always understood, and that developing countries need to consider these issues. The ecohealth approach is useful when exploring this issue.

We highlight three implications of the ecohealth approach: (1) The WHO definition of health as a state of complete physical, mental and social well-being emphasized that health is more than the absence of disease. However, because this approach may involve an unattainable goal, we suggest that health should be defined in the ecosystem context, and the goal should be to attain acceptable and sustainable levels of health through enabling people to realize decent livelihoods, and to pursue their life purpose; (2) The increasing interconnectedness of ecosystems in a globalizing world requires an ethical approach that considers human responsibility for the global biosphere. Here, ecohealth could be a countervailing force to our excessive concentration on economy and technology; and (3) If ecohealth is to become a positive agent of change in the global health promotion movement, it will have to find a secure place in the educational curriculum.

**Summary:**

This article presents a brief case study of Japan’s development assistance to Laos, and its environmental and health implications, as an illustration of the ecohealth approach. We highlight three implications of the ecohealth perspective.

## Background

### Relevance of the ecological perspective on human health

The health promotion movement, as reflected in the Ottawa Charter of 1986 [1] squarely recognized the importance of ecosystems and sustainable resources for human health. The Charter was important in encouraging a move toward a more holistic approach to health, applying whole systems thinking and emphasizing the integration of a commitment to health into the fabric of culture, social structures, processes and routine life in human communities. In practice, the healthy settings approach is mostly applied to organizational environments such as schools and workplaces, as convenient places for health interventions. With roots in similar thinking, a city-based health promotion approach evolved in Western Europe, and was extended to North America and later to other regions of the world. In addition, the WHO’s healthy city concept were promoted in a number of developing counties between 1995 and 1999 [2]. With a strong focus on empowerment and participation, being concerned with individuals’ autonomy and ability to pursue health lifestyles and live a healthy life [3], the healthy city movement provides a practical, integrated approach to health at the individual city level. It is, however, limited to urban settings, and in the developing world may be largely dependent on development assistance.

In this article, we are concerned with conceptualizing and understanding health in its wider environmental or ecosystem context, within a globalizing world. From this perspective it is disappointing that, initiatives such the health settings and healthy cities movements notwithstanding, the broad ecological perspective seems to have faded from the health promotion movement [4]. The recent Bangkok Charter of 2005 [5] and subsequent global health promotion conferences [6,7] appear to confirm this view. Thus, to address the complex health risks associated with globalization and global environmental change, we argue that the ecological framework for understanding human interaction with the world, including values, principles, and ethics, needs to be revived and renewed.

For a long time, developing countries have been struggling with communicable diseases such as malaria, diarrhea, and HIV. Today, these developing countries are faced with the double burden of these diseases, and an increase in the incidence of non-communicable diseases, including cardiovascular diseases, diabetes, and cancer [8]. Traditionally, reductionist approaches were adopted to reduce the burden of disease [9]. Researchers would investigate the major risk factors associated with specific diseases, and devise plausible preventive measures corresponding to each of these where possible. However, risk factors are mostly interdependent, and intertwined with our livelihoods and ecosystems. Reductionist approaches tend to overlook these interdependencies and the trade-offs communities make between human health and their societal, cultural, and ecosystem environments, and it has been pointed out that the resulting solutions often turn out to be only a temporary relief, and can lead to new levels of problems [10,11]. Thus, to address the double burden of disease developing countries are facing, a broad ecological perspective to health is called for.

Ecosystems are the planet’s life-support systems -- for the human species and for all other forms of life. They are interdependent and function as dynamic units [12]. Stable ecosystems and sustainable resources are the fundamental determinants of the health and well-being of the earth’s population [13]. For the young, who make up a quarter of the world’s population, it is even more important to take a long-term perspective and consider the interrelations between ecosystem change and human health [14].

Jared Diamond [15] has recently argued that, historically, the survival or collapse of societies can be explained from the perspective of their interactions with the ecosystem. In other words, the health of the ecosystem determines the survival of individuals as well as of groups, communities, and societies. In the 21st century, the globalization not only of economy and society, but also of environmental change, means that any degradation of ecosystems affects societies and human health globally. Thus, human health absolutely depends on earth’s ecosystems and the services they provide [12]. Conversely, the ecosystem is affected by human livelihood requirements, behavior, and social development. One of the great development challenges for the 21st century is how human communities can avoid compromising human health while meeting growing demands on resources and ecosystem services and at the same time promote thriving, resilient communities and environmental sustainability [16]. Innovative ideas and paradigms, in the “real world” and in research, are needed to address this challenge.

Arguably, improving people’s health and well-being requires changing the way people interact with their natural and built environment, and the findings from innovative research may be able to point the way. Examples of successful research projects suggest that the ecohealth approach can contribute to new scientific knowledge and innovations, reinforce community empowerment and initiatives, and promote policy change [16].

### What are ecohealth and successful ecohealth research?

Up to the present, there is no overall consensus on the definition of ecohealth, but the term conveys both the idea that ecosystems can be healthy or unhealthy, and the notion that people’s health depends on the health of the ecosystem [16].

Epistemologically, the central notion of ecological thinking is interdependent, interactional, and/or reciprocal relationships between social system, development, human health, human livelihoods, and ecosystems. Contrary to traditional ecology, which highlighted the physical and biological features of environment, the ecological approach to health is more social-ecological in nature. It focuses more than before on the social, organizational, and cultural components of the environment; consistent with the Ottawa Charter of Health Promotion [1]. In other words, an ecohealth approach recognizes that human health and well-being are the result of a complex and dynamic set of interactions between people, social and economic conditions, culture, and the natural environment [4]. Where traditional health education strives for individual health behavior modification, ecohealth research argues for more focus on people and the socio-cultural features in ecosystems, and pursues changes in health behavior through participatory processes such as action research. This is research in which researchers and lay people, including community members collaborate to enhance the biophysical, social, and cultural dimensions of the environment: [17].

The ecohealth approach requires systems thinking that is in sharp contrast with the risk-factor analyses that are commonly used in biomedical or epidemiological approaches to health research. Such risk-factor analyses tend to treat individual environmental factors separately by ignoring the interrelationships among them, although epidemiologists are gaining insights into the complexities of dynamic social and environmental systems by thinking about population health in ecological terms [18]. Whereas studying the social determinants of health does not fully address the environmental drivers of inequity or of poor health, the ecohealth approach looks into disparities in quality, vulnerability, and resilience of the ecosystem connections between the poor and the rich. Therefore, this approach, often applied in the context of developing countries, focuses on the interactions between the ecological and sociocultural-economic dimensions of a given situation, and their influence on human health, as well as how people use or impact on ecosystems [16].

As ecohealth research continues to probe such interactions, it has increasingly become clear that modern economic development itself has been at the root of the changes in ecosystems which have in turn affected human health. A central theme of relevance to the contemporary world therefore, is that development strategies imply choices and decisions that have profound health implications. There is a need for this realization to be incorporated more systematically into development planning.

For several years, we have been engaged in collaborative research on malaria and liver fluke infestations in rural areas of Laos, as well as in the development of an ecohealth education curriculum for schools in Laos; in partnership with the National University of Laos and the Lao Ministry of Education. In this article, we briefly examine the experience of Japan in addressing environmental health issues within Japanese Official Development Assistance to Laos, and make the case that ecohealth analysis is a promising approach when considering the challenges faced by developing countries.

## Discussion

### The Japanese experience

“A true civilization does not devastate mountains and rivers, nor does it ruin villages or kill people” [19]. A hundred year ago, Shozo Tanaka (1841–1913), the pioneer of the environmental protection movement, wrote down this monumental remark in his diary while protesting against mineral pollution by the Ashio copper mine. In the 20th Century, according to Japan’s Basic Environment Law, the purpose of environmental conservation is “to ensure healthy and cultured living for both the present and future generations of the nation as well as to contribute to the welfare of mankind” [20].

Japan’s experience in environmental conservation during the pre-modern era, before the Meiji Restoration of 1868, is often regarded as a successful adaptation to ecosystem principles [15]. One often-quoted successful example of Japan’s environmental sustainability approach in the Tokugawa Period was the system whereby human waste (“nightsoil”) from the cities was used as fertilizer in the nearby countryside. It is argued that this lowered the mortality rate in Japanese cities due to diseases such as cholera and dysentery, as compared to American cities [21]. After the Meiji Restoration, however, the national policy of rapid industrialization and economic growth caused severe water and air pollution, nasty odors, loud noises, and an insanitary urban environment in Japan [22]. As many of the new technologies transplanted from developed countries affected the environment seriously, local citizen protest movements against environmental pollution often ensued. The early 20th century saw foresighted humanist pioneers such as Shozo Tanaka fighting against the government as well as companies to protect the environment, and secure human health and livelihood rights.

Japan has learned from its harsh experiences with environmental pollution. The widely known “four big pollution diseases of Japan” (Yokkaichi asthma caused by severe daily exposure to outdoor air pollution in Mie prefecture, Itai-itai disease or chronic cadmium poisoning in Toyama prefecture, Minamata disease and Niigata-Minamata disease or methyl mercury poisoning in Kumamoto prefecture and Niigata prefectures respectively, which resulted from policies that over-emphasized high economic growth and benefits for the heavy and chemical industries [23,24], brought about the introduction of a Basic Environment Law in 1993. With its early recognition of human-induced global environmental changes, and the resulting adverse health consequences [25], this law may be regarded as an advanced set of rules. Nevertheless, modern Japan is hardly worthy of praise as an advanced or successful example of the battle with environment pollution and the prevention of adverse health consequences [22].

The “Japanese economic miracle”, with a growth of GDP per capita from US$1,921 in 1950 to US$18,789 in 1990, was in fact achieved at a tragic cost in public health problems caused by severe environmental pollution. As a result, it is also memorable for the plight of the victims of environmental pollution such as methyl mercury poisoning, which made history by providing the name to the Minamata Convention on Mercury [26]. Over 60 years have passed since this disease was recognized, but lawsuits regarding official certification and compensation of Minamata disease patients have still not come to an end. Moreover, in recent years the Japanese government has appeared to be passive in controlling the pollution from the radioactive materials discharged as a result of the Fukushima nuclear disaster [27,28]. The government still appears to attach greater weight to vested institutional interests and the economic market, than to considerations of environment and health. While several scientific publications have cautioned about radioactive pollution on land and in the sea [29-32], the government is maintaining a “safety myth”, and does not seem to take the threat of pollution of ecosystems by radioactive agents such as Cesium-137 and Xenon-133 on the long-term health of the Japanese population seriously [33,34]. While Japan rapidly increased life expectancy due to rapid economic growth in a relatively egalitarian social structure, and has now achieved the highest level of longevity [35], when faced with the fundamental tradeoff between industrial development and environmental protection, the government chose to sacrifice environmental sustainability and population health. Unfortunately, Japan has not realized its unique potential to apply local experience with pollution-related diseases to the prevention of similar catastrophes in other countries.

In this way, Japan as a developed country cannot serve as a model to developing countries in any direct sense [24,36]; rather, developing countries may have the potential to be models in their own right. The high consumption lifestyle of developed countries, which depends on the low pricing of natural and human resources, would not be realized without the resources of developing countries [9]. Organizations engaging in international cooperation should consider such a perspective. The future of developing countries does not lie in the extension of the logic and culture of the developed countries, but rather must be found somewhere beyond them. In pursuing alternative futures, new ecological concepts and frameworks are necessary to develop models that do not compromise people’s health while they promote resilient communities and environmental sustainability.

### Limitations of Japan’s assistance to Laos

For a long time, Japan has been providing substantial economic assistance for the socioeconomic development of Laos; including development of infrastructure, health care, education and human resources, and assistance for food security and agricultural development. It is important to consider whether the potential of such assistance to contribute to socioeconomic development is realized, or whether it possibly has had a negative impact on the natural and social environment, and on population health in Laos. We should use the lessons learned from Japan’s tragic experiences of local ecosystem destruction and health problems [36] for the purpose of preventing similar problems in Laos.

Laos renounced any World War II reparations from Japan in 1957, and Japan undertook to provide economic and technical support to Laos in an agreement signed in 1958. Japan has continued to assist the country, and to date it is the top Organization for Economic Co-operation and Development donor in Laos. For example, from 2008 to 2012, Japan provided a total of US$1.8 billion, including 92 million as loans, 1.1 billion in grants, and 616 million for technical cooperation [37]. According to a Japanese Ministry evaluation report on development assistance to Laos, Japan invested 46% of its grant aid in the development of social infrastructure. It was also documented in this report that the construction of a hospital, an international bridge, roads, an international airport, and some facilities at the National University of Laos were widely acknowledged by Laotian participants in a small survey [38]. In the period 1997–2003, Japan allocated almost half of its assistance in Laos to transportation infrastructure, which was a higher proportion than other major donors. Construction and maintenance of main roads can make mass transportation of people and materials possible and speedy.

It is true that social development based on such overseas financial assistance can contribute to improving standards of living and modernize the lifestyle of people in developing countries. At the same time though, changes due to socioeconomic development may put a burden on the social and natural environment, and may cause profound changes in ecosystems. Some developments in Laos suggest that this is indeed happening. For example the population has become more concentrated in Vientiane Capital (10.5% out of 2.9 million in 1985 to 12.5% out of 5.6 million in 2005), as have large factories (66% of the total number of large factories), while forest land in Vientiane Capital is being lost (69.2% of land cover in 1995 to 67.5% in 2005). Canals and waterways are also increasingly polluted due to the increase in garbage [39]. Although how much hazardous and toxic waste is generated by industries in total is as yet unknown, it has been estimated that approximately 183 tons of health-care waste per day are produced by hospitals and health-centers in Vientiane, and are mixed with municipal waste for garbage disposal [40]. Also, most of the 112 industries generating wastewater have no wastewater treatment facilities, and discharge directly into rivers [40].

**Figure 1:**
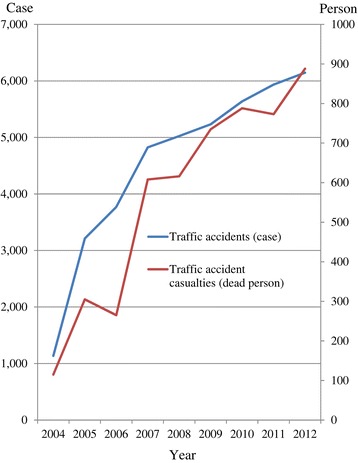
**Traffic accidents and fatal casualties in Laos 2004–2012.** Source: http://www.ajtpweb.org/statistics/Laos/road-transport-of-laos.

In particular, with the first Thai-Lao Friendship Bridge built in 1994 as a start, motorization has accelerated in Laos. The total number of registered motor vehicles increased fourfold from 138,607 in 1995 to 568,290 in 2005 [41]. Thereafter, it continued to increase. Motor vehicles numbered 1.3 million in 2012; probably an underestimation due to the exclusion of taxis and public buses [37,39,42]. Because of the extension of roads and increase of motor vehicles, the number of traffic accidents has also risen, from 1,136 cases in 2004 to 6,146 cases in 2010 (Figure 1). Simultaneously, deaths caused by traffic accidents also increased from 115 to 888 in the same period [43]. Moreover, vehicles and accidents were concentrated in the Capital Vientiane, 46% and 42% respectively in 2008 [44]. Air pollution mainly from automobile sources such as exhaust gas and dust blown up from unpaved roads, or roads under construction, may in turn affect the respiratory health of many people living along roadsides, although overall air quality is considered to be good in the urban and rural areas of this country [40,45]. These changes support the assertion that the health consequences of rapid development of private transportation in Laos should be considered in future planning.

Also, food market globalization paralleled with urbanization has facilitated dietary changes that have increased the daily intake of nutritionally poor but energy-dense and excessively salty foods in this country, in the same way as seen in quite a few middle and low-income countries [46,47]. The emergence of such “obesogenic” milieus is giving rise to an epidemic of non-communicable diseases in developing countries [4,47,48]. A paradox relating to food security in Laos has occurred: development might be expected to improve this, but the reality in terms of the types of food consumed has been far from desirable [48]. Here we argue that ecosystem (ecohealth) approaches to human health are promising as avenues for exploring long-term solutions to the kind of complex (or “wicked”) problems developing countries are facing [49].

On the other hand, in a country where the development of tourism (often labeled “eco-tourism”) is a top priority, the social and natural environment, in addition to cultural heritage, are of crucial importance [50]. Understandably, the “Principles of Lao Ecotourism” are to decrease environmental and cultural impacts from tourism, while maximizing the benefits for the Lao national economy, especially for local businesses and people [51]. From these principles we can derive a future vision of a Laos that not only promotes modernization of social systems and people’s lifestyles in terms of socioeconomic development, but also conserves culture, and the social and natural environment. We would argue that these goals should also be harmonized with the pursuit of sustainable health. Such a vision of Laos is in accord with the philosophy of ecohealth discussed below.

### Three implications of the ecohealth perspective

The concept of ecosystem approach to health is not brand new. It goes back to the 1980s, when the Ottawa Charter, with as its main idea of a socio-ecological approach to health, was released [1], and environmental movements had begun to attract considerable attention [10,52]. In this section, we address a few implications of the values, principles, and ethics of the ecohealth perspective that have not been clearly pointed out yet, in order to enhance recognition of the importance of this framework for understanding our health and the world.

#### A. Implications for the concept of health

The definition of health is very important because it determines the goals of health policy, health services, and health interventions. The often-quoted 1948 WHO definition of health as a state of complete physical, mental and social well-being served powerfully to underscore that health is not just the absence of disease. At the same time, the level of ambition reflected in this formulation means that under many circumstances such health levels may be an unattainable goal. Recently, the British Medical Journal [53,54] pointed out that this definition may have unintentionally promoted the medicalization of society, as more and more human characteristics were identified as risk-factors for human health. It suggested that, under the conditions of rapid societal ageing in the developed world, a better definition would refer to the ability to adapt and self-manage in the face of personal social, physical, and emotional challenges.

In addition to this challenge of redefining health in ageing societies, our view is that the level of human health that can be attained is subject to the capacity of the ecosystem. In other words, we think that, from an ecohealth perspective, human health is determined by the quantity and quality of goods and services from the ecosystems within which people live and engage in their livelihoods. Over-exploited ecosystems cannot sustain healthy human livelihoods, and can be hazardous to human health. Accordingly, we propose a concept of health that takes into account the constraints of the wider ecosystem, where the goal is to attain acceptable levels of health that are sustainable, and enable people to realize decent livelihoods and pursue their life purpose. In this view, living healthily within the constraints of the ecosystem implies a degree of adaptation and self-restraint that resonates to some degree with the above definition of health as the ability to adapt and self-manage.

This view of health as being embedded in ecosystems needs however to be qualified in two ways. First, in a globalizing world, the scale and scope of “ecosystem” needs to be specified for the discussion of ecohealth to gain traction. In some situations, the limited or degraded endowments in the local ecosystem may be such that maintaining health at levels needed for realizing livelihoods and life purpose is extremely challenging^a^. Increasingly, however in the present globalizing world, local ecosystems are more often than not linked with others in a multitude of ways. This means that human health is not only embedded in local ecosystems, but can benefit or suffer from connections to other places and systems. And as humanity pushes the limits on a planetary scale, global environmental change is becoming a more important factor in human health.

Second, an ethical dilemma exists in that it is not only different resource and service endowments across ecosystems that constrain or facilitate health, but also that not all people have equal access to such endowments, given the dynamics of power, marginalization, and social stratification in many societies. Clearly, eradication of poverty and social development are crucial factors in improving human health in the developing world. However, as the above examples of Japan and Laos suggest, resolving health disparities through the improvement of socioeconomic conditions may accelerate degradation of the environment (e.g. “Grow Now, Clean Up Later” [36]). To address this vicious cycle is a challenge, and sociocultural changes to underpin an emphasis on the higher value of quality of life will be indispensable along with advancing social development in solving this problem. This is discussed in the next section.

#### B. Implications for ethical relevance

It is now inevitable that humanity needs to search for effective sustainability strategies that take into account our survival over the next hundreds to thousands of years. As part of this, we need to enhance humanity’s attitude towards living together with the natural environment, strengthen positive attitudes to learning from the environment, and the gaining of wisdom and knowledge about the ecosystem, and ultimately the values that those attitudes are based on. Accordingly, the more ecosystems and the human livelihoods in them are changing, the more an ethic of development is required that considers human responsibility for the global biosphere [55-57].

However, in reality, an overwhelming emphasis on economy and technology over human responsibility to the ecosystem or eco-ethics still prevails in the modern world; in both developed and developing countries. Obviously, in our argument for change we do not deny the potential for positive social development from the introduction of new technology. We also support positive economic development where globalization helps meet the increasing needs of human livelihoods. Nonetheless, the excesses seen in the past and present will bring about the further degradation of ecosystems, and a subsequent deterioration of human health, if they are allowed to continue unchecked.

Since ecohealth is an approach that places high value on harmonious and sustainable relationships between the needs of human livelihoods, ecosystems and human health, it naturally follows that people should run their livelihoods by considering the dynamic nature of the ecosystem. Ethically, this idea is in more accord with a sustainability approach which strives for a good balance between homocentric and eco-centric perspectives [56]. Therefore, ecohealth could inspire a countermeasure or counter culture against the excessive valuing of economy and technology. We therefore think ecohealth is an ethically more relevant approach to health in the developing as well as in the developed world.

#### C. Ecohealth education

While it is important to avoid environmental determinism, ecohealth education is key for enhancing people’s environmental awareness, which in turn helps to motivate or encourage them to maintain a harmonious and sustainable relationship between the ecosystem and activities for livelihoods, communities and health [24]. Ecohealth education in school is particularly important because it is the social responsibility of adults to educate children and young people to become positive agents of change for ecosystem sustainability, social development, and human health.

Some of the significant features of ecohealth as a research approach are community participation, action research, and systems thinking. In ecohealth research collaborations, researchers and community residents explore practical solutions to problems in the community, as a team. Although community residents are often regarded mostly as laypersons from a medical research perspective, they have substantial knowledge and wisdom relating to local or neighboring ecosystems, livelihoods and behavior, and they are the primary stakeholders in any research activity that happens in their locality. Successful examples of the use of local knowledge in finding solutions to local health problems have been documented [10,16,58]. In the view of these studies, collaborative action research in which researchers and community members work closely together is an essential element of the ecohealth education approach.

## Summary

In considering the health and environmental problems developing countries like Laos are facing, the past experiences of Japan are relevant not so much as a model to follow, but rather as a cautionary tale that identifies that an excessive emphasis on rapid economic development can come at a heavy cost in terms of environmental degradation and serious population health issues. We argue that a holistic ecohealth approach, which asks questions about the fundamental drivers of environmental changes and their health consequences will, by examining the interrelationships among the elements of ecosystems, provides a promising approach for exploring and addressing these issues.

Such an ecohealth approach entails a concept of health that takes into account the constraints of the wider ecosystem, aiming to attain acceptable levels of health that are sustainable, and to enable people to realize decent livelihoods, and pursue the purpose of their lives. In a globalizing world, “the ecosystem” is also increasingly worldwide, and an ethical approach that emphasizes human responsibility for the global biosphere is needed. If ecohealth is to gain prominence in the global health promotion movement and provide a counterbalance to short-term growth maximization strategies though, it will need to find a place in the educational curriculum to stimulate young people to become positive agents of change. Japan, as a major donor in Laotian development, has the responsibility to monitor the environmental and health impacts of its assistance, and should take the initiative to provide education based on ecohealth thinking to citizens and children.

## Endnote

^a^E.g. for a discussion of the impact of poor ecosystems conditions on health in Africa, see [59].
